# Trends in educational inequalities in old age mortality in Norway 1961−2009: a prospective register based population study

**DOI:** 10.1186/1471-2458-12-911

**Published:** 2012-10-27

**Authors:** Joakim Oliu Moe, Ólöf Anna Steingrímsdóttir, Bjørn Heine Strand, Else-Karin Grøholt, Øyvind Næss

**Affiliations:** 1 Department of Health Management and Health Economics, Institute of Health and Society, Faculty of Medicine, University of Oslo, Blindern, P.O. Box 1089, Oslo, 0317, Norway; 2 Division of Epidemiology, Norwegian Institute of Public Health, Nydalen, P.O. Box 4404, Oslo, 0403, Norway

**Keywords:** Social inequality, Mortality differentials, Health trends, Old age, Nordic welfare model

## Abstract

**Background:**

The vast majority of deaths occur in older adults. Paradoxically, knowledge on long-term trends in mortality inequalities among the aged, and particularly for those aged 80 years and over, is sparse. The historical trends in size and impact of socioeconomic inequalities on old age mortality are important to monitor because they may give an indication on future burden of inequalities. We investigated trends in absolute and relative educational inequalities in old age mortality in Norway between 1961 and 2009.

**Methods:**

We did a register-based population study covering the entire Norwegian population aged 65-94 in the years 1961−2009 (1,534,513 deaths and 29,312,351 person years at risk). By examining 1-year mortality rates by gender, age and educational level we estimated trends in mortality rate ratios and rate differences.

**Results:**

On average, age-standardised absolute inequalities increased by 0.17 deaths per 1000 person-years per year in men (P<0.001), and declined by 0.07 deaths per 1000 person-years per year in women (P<0.001). Trends in rate differences were largest in men aged 75−84 years, but differed in direction by age group in women. The corresponding mean increase in age-standardised relative inequalities was 0.4% and 0.1% per year in men and women, respectively (P<0.001). Trends in rate ratios were largest in the youngest age groups for both genders and negligible among women aged 85−94 years.

**Conclusions:**

While relative educational inequalities in old age mortality increased for both genders, absolute educational inequalities increased only temporarily in men and changed little among women. Our study show the importance of including absolute measures in inequality research in order to present a more complete picture of the burden of inequalities to policy makers. As even in older ages, inequalities represent an unexploited potential to public health, old age inequalities will become increasingly important as many countries are facing aging populations.

## Background

Life expectancy has been increasing continuously in developed countries, leading to an ageing of societies [1]. Since the 1950s, this development has been driven increasingly by declining mortality among the aged [2]. However, the benefits of this trend have not been evenly distributed, as population figures conceal underlying socioeconomic inequalities in mortality trends [3].

From an earlier study we found that educational inequalities in mortality among Norwegians aged 45−64 years increased in the period 1960 to 2000 [4]. The corresponding widening of inequalities in life expectancy at age 35 seems to have resulted from delayed onset of gains in life expectancy in lower-educational groups compared with higher-educational groups [5]. Knowledge on trends in mortality inequalities among the aged, and particularly for those aged 80 years and over, is sparse, yet paradoxically, the vast majority of health problems, health services consumption, and deaths occur in older age groups [6,7]. Most of the published studies on trends in inequalities among older adults [3,8-15] have been restricted in age [3,8,15] or were conducted after 1980 [3,8-10,15]. Although some studies covered the period before 1980 [11,12,14] or after 2000 [10], or assessed temporal shifts in trends [8,11,13], there is a lack of studies of trends and their nuances over prolonged periods. Further, some studies have relied on relative inequality measures without notions of trends in mortality or absolute mortality differentials [9,12,13,16].

The choice of inequality measures in old age groups is particularly important because health problems tend to increase with age and policymakers need to anticipate increasing or declining inequalities in old age in the future. Although different inequality measures express different dimensions, the rationale for choosing one measure over another is rarely the subject of reflection [17]. Rate ratios (RRs) that express the relative scale of inequalities in health are the traditional choice in aetiological investigations [16,18]. They are scale-neutral, and therefore useful for directly comparing rates of outcomes measured on different scales [16,19]. Scale neutrality also implies that using ratios alone implicitly endorses equality *per se*, independent of the level or direction of trends in group-specific or overall population health [17]. Thus, rate ratios conceal the burden of inequalities [20] and thereby their importance to population health [16,17]. Public health importance might be better expressed in terms of rate differences (RDs) because they quantify the excessive rates attributable to being in the disadvantaged group of interest [17,21-23].

The relative risk of lower compared to higher socioeconomic position tends to diminish with age [6,12,24]. Some researchers have explained this tendency with health selection as being due to particularly high premature mortality in disadvantaged groups [7,24,25], or postponement of ill-health in groups of higher socioeconomic position [26]. However, age itself is a powerful risk factor for death [27,28]. The risk for death increases exponentially with increasing age and dominates most other risk factors for old age mortality [29,30]. Thus, in higher ages, we might expect the relative effects of risk factors such as low socioeconomic position to be more stable over time than in younger ages. The apparently negligible scaling and trends in relative inequalities in old age mortality conceal the fact that the largest mortality rate attributable to lower education occurs in the oldest age group. Thus, trends in absolute inequalities are particularly relevant when studying old age mortality [6].

Inequalities in health can be considered unfair [31,32] and a violation of the fundamental human right to enjoy the highest attainable standard of lifelong health [33]. However, overcoming inequalities is considered an achievable prevention strategy [32] that has enormous potential for improving population health [31,34]. Even in older ages, mortality and ill-health is amenable to interventions [35] and the interventions do not have to be lifelong [36]. Demographic studies provide strong evidence that changes in a wide range of current conditions are important for old age mortality, even for octogenarians and nonagenarians [36]. Numerous prevention strategies have proven to be effective in older ages [37-48] and treatment benefits might be more marked in older than younger individuals due to their greater risk of diseases and death [46]. Nonetheless, many of the potentially beneficial prevention strategies are probably not fully exploited. In addition, organizational features might also be of importance. For instance, fee-for-service financing might favor cure more than prevention [49], and lack of integration between hospitals and primary health care [50] might hamper effective treatment and rehabilitation of frail elderly. As ill-health can be prevented and death be delayed, old age inequalities in health should also be possible to reduce.

We investigated trends in relative risk (rate ratios) and absolute risk (rate differences) of educational inequalities in old age mortality in Norway in the period 1961 to 2009 during which considerable changes in mortality, health policy, and expansion of a comprehensive welfare state occurred.

## Methods

### Design, setting and data

Statistics Norway used unique personal id-numbers to link the Central Population Registry to educational data from the 1960 census, and from 1970, with data from the National Educational Database (NUDB). Thus, educational level in the 1960s is classified according to the 1960 census, while educational level after 1970 is classified according to the NUDB. NUDB was created in 2002 and is based on self-reported data in the 1970 census and thereafter of annual administrative records of data on new achievements from all educational institutions in Norway [51].

We generated 49 cohorts defined by all registered Norwegian citizens aged 65−94 years at any time within each year between 1961 and 2009. The 1961–1970 cohorts were restricted to people registered in the 1960 census. Cases that emigrated between 1961 and 1970 were censored in 1965. Cases that emigrated between 1971 and 2009 were included every year up to the time of emigration and excluded thereafter. The cohorts were followed up for deaths occurring within the following year in Norway.

The study was approved by the Regional Committee for Medical and Health Research Ethics, South East Norway (approval number 2010/260).

### Variables

All deaths in Norway are reported on a medical death certificate issued by a medical practitioner and registered in the Central Population Registry. We classified education into two levels. Lower education was defined as compulsory primary or lower-secondary education corresponding to the International Standard Classification of Education (ISCED97) levels 0−2 [52]. Higher education was defined as upper-secondary, post-secondary or tertiary education corresponding to ISCED97 levels 3−6. Although educational data from the 1960 census were coded differently than in the NUDB, we were able to adjust educational level for the 1960s by comparing frequency tables of education codes in 1960 and 1970. Adjustment was done by recoding all person-years in the 1960s according to the most frequent transition in educational classification between the 1960s and the 1970s. Education was almost complete for all cohorts. The highest proportion of missing educational data (both genders, total) was 2.6% in 1969 and 1970 (Additional file 1: Table A1). The trend effects were given by the continuous variable “year” which was equal to calendar year minus 1960.

### Statistical analysis

We calculated age-standardised and age-specific mortality rates for educational groups separately by gender. Age-standardisation used the direct method with the mean number of person-years in the operational gender and age groups as the standard population. The population distribution in the 65−74, 75−84, and 85−94 age groups by gender was 61%, 32% and 7% for men, and 54%, 36% and 11% for women, respectively.

We started by estimating the age-standardised and age-specific mortality RD and mortality RR by educational level in both genders, with the higher educated as the reference group. We then tested for trends in mortality rates using Poisson regression models stratified by gender, age group, and educational level, with death count as the outcome variable, person-years as the exposure variable, and year as the covariate. Finally, we tested for trends in relative and absolute educational mortality inequalities in models stratified by age group and gender. Trends in relative inequalities were tested using Poisson regression models with death count as the outcome variable, person-years as the exposure variable, and year, educational level, and the interaction term education by year as covariates. The interaction terms were interpreted as the mean yearly relative changes in RRs. To test for trends in absolute inequalities, we used a weighted least square (WLS) regression model with mortality rate as the outcome variable, and year, educational level, and the interaction term education by year as covariates. The weights were equal to the number of person-years at risk in each stratum. We interpreted the interaction terms as the person-years weighted mean yearly absolute changes in RDs. The overall trends from 1961 to 2009 and a time-restricted analysis of the first and second half of the period were tested separately. Cases with missing values (education) were excluded from the analysis. We controlled for a possible misclassification bias due to differences in educational classification by means of a sensitivity analysis in which we estimated trends in mortality inequalities limited to the years 1971−2009.

We regarded two-sided P values <0.05 as statistically significant and used IBM SPSS Statistic 19 (IBM Corp, New York, USA 2010) for data preparation and analysis by WLS-regression models. Stata/IC 12.0 (StataCorp LP, Texas, USA, 2012) was used for tests using Poisson regression models and for graphics.

## Results

In total, we included 29,312,351 person-years at risk and 1,534,513 deaths in the period 1961−2009 for ages 65−94 years (Additional file 2: Table A2). The proportion of person-years with lower education decreased from 77% in 1961 to 62% in 1981, 43% in 2001 and 33% in 2009 for men (Additional file 3: Table A3). The corresponding decrease for women was from 87% to 70%, 55% and 45% (Additional file 3: Table A3).

**Figure 1:**
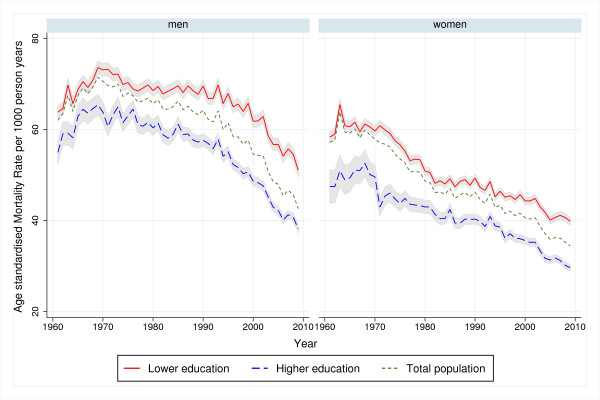
**Trends in age-standardised mortality rates by educational level, 1961−2009. **Men and women aged 65-94 years. One year mortality. Shaded area represents 95% confidence interval.

**Figure 2:**
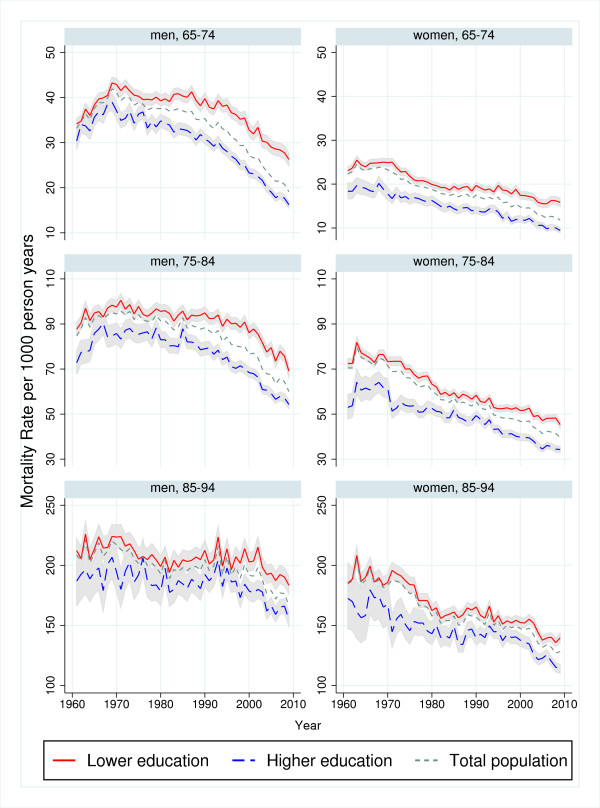
**Trends in age-specific mortality rates by educational level, 1961−2009.** Men and women aged 65−94 years. One year mortality. Shaded area represents 95% confidence interval. Note dissimilar scales on the vertical axis.

**Figure 3:**
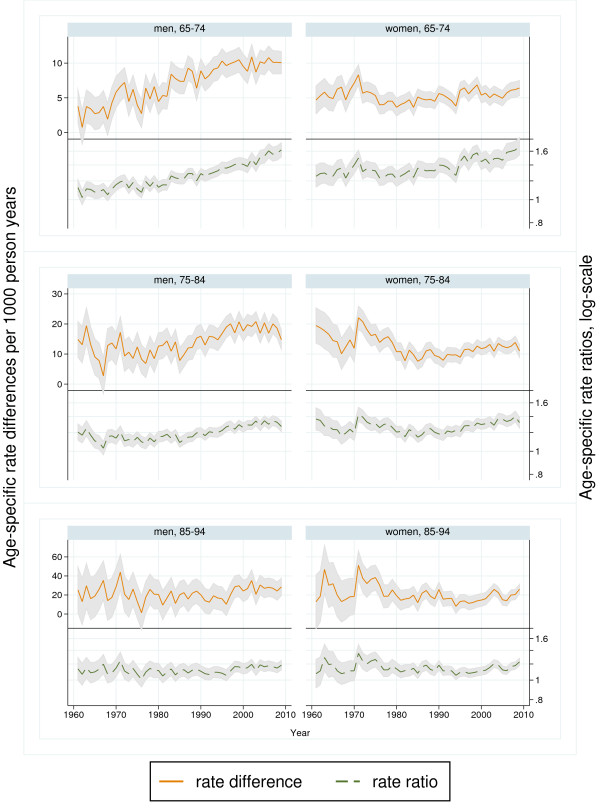
**Trends in rate differences and rate ratios between higher- and lower-educated, 1961−2009.** Men and women aged 65−94 years. Shaded area represents 95% confidence interval. Note dissimilar scales on the vertical axis.

Between 1961 and 2009, the mortality for men declined in all age and educational groups (P<0.001), but the trends shifted over time (Figures 1 and 2). In both educational groups, mortality initially increased or remained unchanged before it started to decline. The decline appeared to start 5−10 years later among lower-educated than higher-educated men (Additional file 4: Table A4, Figures 1 and 2). Educational RDs and RRs increased in all age groups (Table 1). Between 1961 and 2009, the weighted mean yearly increase in the age-standardised RD was 0.17 per 1000 person-years and the increase was largest among men aged 75−84 (Table 1a). The corresponding mean yearly increase in age-standardised RR was 1.004 and the increase was largest among the younger age groups (Table 1b). The time-restricted analyses show that the increases in RDs and RRs were for all age groups mainly in the period 1985−2009 (Table 1). Both RDs and RRs increased in men aged 65−74 years in the period 1961−1984, and in all age groups thereafter (Table 1), but the increase seems to slow down after the 90s (Figure 3).

**Table 1:** Trends in educational inequalities in mortality, men and women aged 65−94 years between 1961 and 2009

**a) Temporal trends, mean yearly absolute change in RD (95% CI). P value**						
*Gender, age*						
*Men*	1961−1984		1985−2009		1961−2009	
65−74	0.22 (0.04 to 0.40)	0.018	0.14 (0.06 to 0.23)	0.001	0.28 (0.19 to 0.36)	<0.001
75−84	0.06 (−0.24 to 0.37)	0.675	0.39 (0.18 to 0.60)	<0.001	0.38 (0.23 to 0.53)	<0.001
85−94	−0.26 (−1.01 to 0.49)	0.492	0.68 (0.06 to 1.31)	0.034	0.31 (0.04 to 0.58)	0.025
*Age standardised*	0.07 (−0.05 to 0.19)	0.263	0.21 (0.13 to 0.29)	<0.001	0.17 (0.11 to 0.24)	<0.001
*Women*						
65−74	−0.08 (−0.17 to 0.02)	0.134	0.064 (0.02 to 0.11)	0.010	0.01 (−0.02 to 0.03)	0.740
75−84	−0.26 (−0.58 to 0.05)	0.095	0.18 (0.08 to 0.28)	0.001	−0.10 (−0.17 to −0.02)	0.013
85−94	−0.36 (−1.27 to 0.56)	0.438	0.26 (−0.17 to 0.69)	0.232	−0.16 (−0.40 to 0.08)	0.186
*Age standardised*	−0.17 (−0.27 to −0.07)	0.001	0.10 (0.06 to 0.15)	<0.001	−0.07 (−0.10 to −0.04)	<0.001
**b) Temporal trends, mean yearly relative change in RR (95% CI). P value**						
*Men*	1961−1984		1985−2009		1961−2009	
65−74	1.006 (1.004 to 1.008)	<0.001	1.012 (1.011 to 1.014)	<0.001	1.011 (1.010 to 1.011)	<0.001
75 to 84	1.001 (0.999 to 1.002)	0.445	1.008 (1.007 to 1.010)	<0.001	1.006 (1.005 to 1.006)	<0.001
85 to 94	0.999 (0.996 to 1.002)	0.506	1.004 (1.003 to 1.006)	<0.001	1.002 (1.001 to 1.003)	<0.001
*Age standardised*	1.001 (1.000 to 1.002)	0.002	1.007 (1.006 to 1.008)	<0.001	1.004 (1.004 to 1.004)	<0.001
*Women*						
65 to 74	1.000 (0.997 to 1.002)	0.752	1.009 (1.007 to 1.011)	<0.001	1.005 (1.004 to 1.005)	<0.001
75 to 84	0.998 (0.996 to 1.001)	0.155	1.007 (1.006 to 1.009)	<0.001	1.002 (1.001 to 1.002)	<0.001
85 to 94	0.999 (0.996 to 1.002)	0.684	1.003 (1.002 to 1.004)	<0.001	1.000 (0.999 to 1.001)	0.821
*Age standardised*	0.999 (0.998 to 0.999)	<0.001	1.005 (1.004 to 1.006)	<0.001	1.001 (1.000 to 1.001)	<0.001

a) Mean trends in age-specific and age-standardised mortality rate differences (RDs) per 1000 person-years between higher- and lower-educated men and women, 1961−2009. Estimated by use of weighted least square regression using weights equal to person-years at risk in each strata. b) Mean trends in age-specific and age-standardised mortality rate ratios (RRs) between higher- and lower-educated men and women, 1961−2009. Estimated by use of Poisson regression.

The mortality of women also declined in all age and educational groups between 1961 and 2009 (P<0.001) but shifted during the study period (Figures 1 and 2). For women, the decline was more constant and with a more similar absolute pace in the two educational groups compared with men. However, an apparent convergence of mortality during the first two decades seems to have preceded a period of slightly diverging trends. Educational RDs decreased among women aged 75−84 in the period 1961−2009, while there were no significant overall trends in RDs among women aged 65−74 and 85−94 (Table 1a). This resulted in an overall weighted mean yearly decrease in age-standardised RDs of −0.07 per 1000 person-years (Table 1a). Trends in educational RRs increased except during the 1970s, with an overall mean yearly increase in age-standardised RRs of 1.001 (Table 1b). The largest relative changes occurred among the youngest age group while the trend was not statistically significant among women aged 85−94 (Figure 3). According to the time-restricted analyses and Figure 3, both RRs and RDs appeared to decrease during the 70s, stabilize during the 80s and increase during the 90s. However, there were no statistically significant age-specific trends during the first half of the observation period, in neither absolute nor relative inequalities (Table 1). In the period 1985−2009, RDs and RRs increased slightly in all age groups, except for the trend in RD for those aged 85−94, which was not statistically significant. In both genders, neither the age-standardised trends nor the age-specific trends in inequalities differed substantially in significance level or direction from trends in the sensitivity analysis limited to the years 1971−2009 (Additional file 5: Table A5).

## Discussion

### Key findings

During the last five decades all groups have benefited from declining mortality, but not equally; absolute educational inequalities periodically increased in men but changed little in women, while relative inequalities increased in both genders. The increase in absolute inequalities slowed down in men in the last decades, but is ongoing in the youngest women. While trends in relative inequalities were smaller in older compared with younger age groups, trends in absolute inequalities were largest in men aged 75−84 years.

### Strengths and limitations

This study has several strengths. First, it includes the total target population with very few missing cases. Second, the study period is long, covering almost half a century, which featured establishment of a comprehensive welfare state and high economic growth. Third, by estimating 1-year age-specific mortality, we were able to describe more nuances for the continuous change in educational inequalities than has previously been reported among older persons. Fourth, we illustrated changes in the impact of educational inequalities over decades of substantial increases in educational attainment. Finally, our results are relevant to others, as the Norwegian population resembles other northern European countries in terms of trends in life expectancy and educational attainment [53].

We also note some limitations to this study. First, the different data source for educational level in the 1960s involved potential misclassification. We adapted the educational level from the 1960s to the classification used after 1970. Adjustment error might explain the increased inequalities in mortality at 1971. According to the sensitivity analysis, the trends in mortality inequalities did not change substantially when limiting the study period to 1971−2009 and the effect of this potential misclassification bias is likely to be limited.

Second, most of the older population attained only compulsory basic schooling. As the extent of differentiation in educational level was limited, we compressed the educational hierarchy into two strata. Although this is a rough classification, it reduces the extent of misclassification bias to a minimum and provides results that can be applied in other countries.

Third, the registrations of educational level were self-reported in the 1960- and 1970 censuses, whereas updated data were collected directly from educational institutions thereafter. As the majority of the study population had probably attained their highest education prior to 1970, most of the education level data was solely based on the self-reported data. Although the direction of a possible misclassification bias due to inaccurate self-reporting of educational level is not clear, we find it unlikely that it would substantially bias the trends.

### Comparison with previous studies

Although our main findings are in line with those from other Western countries, they provide more nuanced trends of wider dimensions of inequality than have previously been reported. As mortality increases with age, relative inequalities attenuate whereas absolute inequalities seem to increase [6,12,24]. In most Western countries, old age mortality has declined in all socioeconomic strata over recent decades, but more so among those in higher socioeconomic strata [3,8-11,13]. The same tendency is found in middle-aged populations in Norway [4,54] and other Western countries [3]. According to our analysis, educational inequalities were somewhat weaker in the 1990s compared with earlier analysis of Norwegian data by Huisman et al [6]. The differences are probably due to the use of a different classification of educational level and the use of weights.

Recent gains in life expectancy have been driven primarily by declining mortality among the aged [2]. Thus, the delayed mortality decline among lower-educated older men probably explains most of the corresponding educational lag in life expectancy gain at age 35 that we found in a previous study [5].

### Explanation and interpretation of results

The differences we found between trends in absolute and relative inequalities in mortality are not contradictory, but rather reflect different dimensions of inequalities [17,21,23]. Whereas the largest mortality rate *attributable to* lower education occurs in the oldest age group [6], the corresponding *relative effects* of lower education diminish compared with the overall death risk of old age itself [6]. When viewed over time, the postponement of death to increasingly higher ages allowed the relative effect of risk factors other than age to emerge in the younger age groups. Hence, relative educational inequalities increased. These increases might not be a surprise since proportional mortality declines are unlikely in periods of large mortality declines [54,55]. On the other hand, mortality attributable to lower education increased or remained stable despite major improvements in population health.

The periods of stable absolute inequalities might reflect the accumulated effect of multiple disadvantaged factors associated with lower education. The distribution of causes of death are increasingly diversified with higher ages [56]. While premature mortality tends to affect high-risk individuals and often is associated with certain risk factors, older adults are vulnerable to multiple diseases due to the aging-related physiological functional decline and loss of reserve [56]. Thus, while in younger populations, inequalities in all-cause mortality are sensitive to inequalities in specific risk factors for dominating causes of death, the influence of inequalities in a single risk factor attenuate with higher ages. Hence, the periods of stable absolute inequalities in our study might suggest that the disadvantages of being lower educated persisted regardless of the changes in the prevalence of diseases and known risk factors [57,58].

Nevertheless, there were periods of lagged mortality decline in lower-educated men compared with higher-educated men aged 65−84. This might be interpreted in light of Victora’s reverse equity hypothesis [55] as traces of an education-dependent lag in adoption of major healthy innovations. In this case, such innovations must have been aimed at risk factors that were particularly lethal and common in men. For instance, steeper educational inequalities during the smoking epidemic [59,60] might explain the widening of absolute educational inequalities in mortality among men in our study [61]. This would be in line with findings in studies on inequalities and cause-specific mortality in middle-aged populations, where cardiovascular mortality has been the main driver of socioeconomic diverging trends in mortality [3,4].

In women, the long-term absolute and parallel declines in mortality reveal few dynamics attributable to changing educational inequalities in lethal epidemics. Trends might be shifting, though, as absolute inequalities increased slightly from the 1990s in the youngest age groups. The delayed and weaker dynamics compared with men might be explained by gender differences in smoking history as the proportion of current smokers peaked later in women than in men, and their tobacco consumption was smaller [59].

Another often-mentioned explanation might be that lower-educated people are more homogeneously disadvantaged now than before [3,10,11,58]. A more marginalised group of lower-educated people might be less susceptible to public health initiatives and present an increasing challenge to public health policies. The lower educated group was however not marginal in size during the study period, and constituted one third of all men and almost half of all women in 2009. Compositional changes in the higher educated group might also explain the persistent and widening inequalities. During the study period, the share of tertiary educated within the group of higher educated in our study increased. If there is a mortality gradient by each level of education, this would lead to widening of inequalities in mortality between the two educational groups studied. A rise in educational level within the higher educated group might also explain why the decline in mortality among those with higher education did not slow down as the group grew in size. Instead, they experienced the strongest decline in mortality. Nevertheless, contrasted with the others, mortality was persistently elevated in the group of lower educated people.

## Conclusions

In this study, following the whole old age Norwegian population for almost five decades, we found decreasing mortality in all educational groups, but absolute educational differences in mortality were large and persisting. Also, relative disadvantages in older adults with lower education increased almost continuously as population health improved. Thus, educational inequalities in old age mortality have posed an important and persistent challenge to public health. Inequality research should always include absolute measures in order to present a more complete picture of the burden of inequalities to policy makers. This need is particularly evident among the old. As even in older ages, inequalities represent an unexploited potential to public health, old age inequalities will become increasingly important as many countries are facing aging populations.

## Abbrevations

RD: Risk Difference; RR: Risk Ratio; ISCED97: International Standard Classification of Education; WLS: Weighted least square; CI: Confidence Interval.

## Competing interests

The authors declare that they have no competing interests.

## Authors’ contributions

All authors contributed to the design of the study, interpretation of the results and the editing of the manuscript. Data were compiled by ØN and Statistics Norway. Data were analysed by JOM. All authors read and approved the final manuscript.

## Pre-publication history

The pre-publication history for this paper can be accessed here:

http://www.biomedcentral.com/1471-2458/12/911/prepub

## Supplementary Material

Additional file 1 **Table A1.** Person-time at risk, numbers and percentage of gross data, with missing educational data, by age, gender and educational level in Norwegian men and women aged 65-94 at start of follow-up, 1961-2009. Click here for file

Additional file 2 **Table A2.** Person-time at risk and number of deaths by age, gender and educational level in Norwegian men and women aged 65-94 at start of follow-up, 1961-2009. Click here for file

Additional file 3 **Table A3.** Person-time at risk, proportions, by age, gender and educational level in Norwegian men and women aged 65-94 at start of follow-up, 1961-2009. Click here for file

Additional file 4 **Table A4.** Mortality rate (MR) per 1000 person years by age, gender and educational level in Norwegian men and women aged 55-94 at start of follow-up during 1961-2009. Click here for file

Additional file 5 **Table A5. **Sensitivity analysis. Trends in educational inequalities in mortality, men and women aged 65-94 years between 1971 and 2009. Click here for file
